# Can Electronic Tools Help Improve Nursing Home Quality?

**DOI:** 10.5402/2011/208142

**Published:** 2011-10-10

**Authors:** Kjell Krüger, Line Strand, Jonn-Terje Geitung, Geir Egil Eide, Anders Grimsmo

**Affiliations:** ^1^Løvåsen Teaching Nursing Home, Municipality of Bergen, Løvåsen 26, 5145 Fyllingsdalen, Norway; ^2^Medical Faculty, University of Bergen, 5020 Bergen, Norway; ^3^Haraldsplass Hospital, 5009 Bergen, Norway; ^4^Centre for Clinical Research, Haukeland University Hospital, 5021 Bergen, Norway; ^5^Research Group on Lifestyle Epidemiology, Department of Public Health and Primary Health Care, 5021 Bergen, Norway; ^6^Department of Public Health and General Practice, Norwegian University of Science and Technology, 7491 Trondheim, Norway

## Abstract

*Background*. Nursing homes face challenges in the coming years due to the increased number of elderly. Quality will be under pressure, expectations of the services will rise, and clinical complexity will grow. New strategies are needed to meet this situation. Modern clinical information systems with decision support may be part of that. *Objectives*. To study the impact of introducing an electronic patient record system with decision support on the use of warfarin, neuroleptics and weighing of patients, in nursing homes. *Methods*. A prevalence study was performed in seven nursing homes with 513 subjects. A before-after study with internal controls was performed. *Results*. The prevalence of atrial fibrillation in the seven nursing homes was 18.8%. After intervention, the proportion of all patients taking warfarin increased from 3.0% to 9.8% (*P* = 0.0086), neuroleptics decreased from 33.0% to 21.5% (*P* = 0.0121), and the proportion not weighed decreased from 72.6% to 16.0% (*P* < 0.0001). The internal controls did not change significantly. *Conclusion*. Statistics and management data can be continuously produced to monitor the quality of work processes. The electronic health record system and its system for decision support can improve drug therapy and monitoring of treatment policy.

## 1. Background

Nursing homes face challenges in the coming years due to an increased number of elderly. Quality will be under pressure, expectations of the services will rise, and clinical complexity will grow. New strategies are needed to meet this situation. Modern clinical information systems with decision support may be part of that.

### 1.1. Clinical Issues

Based on clinical experience and information about 513 long-term patients, we believed that there could be failure of the blood-thinning treatment of patients with atrial fibrillation. Likewise, we thought that weighing procedures were followed up for the poor. Use of neuroleptics for the oldest patients is under continuous debate. Therefore, we decided to focus on endpoints within these fields.

One of the most frequent diagnoses among the elderly is atrial fibrillation. Atrial fibrillation is an independent risk factor for developing a stroke [1], and in the elderly it is one of the most important causes of stroke [2]. The current guidelines in force are that all over-75s with atrial fibrillation are to be anticoagulated, but increasing age often produces the opposite effect with decreased prescriptions to the oldest [3, 4].

The presence of nutritional failure in Norwegian nursing homes has been questioned [5]. Little research in the field has been done so far. There is good evidence showing that inappropriate weight loss in the elderly materially impairs their quality of life and life prospects. The convalescence period for illness also increases substantially [6]. A significantly increased risk has been shown for secondary complications like pressure sores [7], infection, depression, and functional impairment [8, 9] as well as increased mortality [10–14].

The high consumption of psychopharmaceuticals in nursing homes has been emphasized in a number of studies. 23% of the demented nursing home population in Bergen was found to be on antipsychotics. The prevalence of psychoactive drugs has been well documented earlier, but some years ago [15, 16]. Double-blind studies have shown little symptom reduction connected with the use of antipsychotics as compared with placebo [17, 18].

### 1.2. Decision Support

 There are weaknesses involved in introducing consensus-based therapeutic regimes in a population. Often test results are not followed up. In a meta-analysis, Kawamoto and colleagues dealt with studies on the effectiveness of decision-making support tools and found that 68% of the systems showed significant improvement in practice [19].

A clinical decision support system (CDSS) is an application that analyzes data to help healthcare providers make clinical decisions. Decision-making support is closely correlated with structured record information, because the decision-making support tools have to respond to correct information in the record—for example, pathological test results. This is easier to accomplish if the underlying data are structured and standardized. A number of major European projects hinge on this issue [20]. 

The traditional medical approach focuses on the individual, with particular emphasis on symptoms, signs and test results emerging from the encounter with individual patients. In order to ensure accessible and correct treatment for all patients, it is also possible to operate using population-directed control measures, for example, keeping track of the proportion of a patient population on a specific drug. This is especially applicable in nursing homes where about 75% of patients are demented and cannot take responsibility for their own followup. The benefit of population-targeted intervention has also been proved in several studies [21].

The users' views on data systems are important for the real-term prospects of disseminating such tools. Doctors appreciate structured recording [22, 23]. Nurses' views on electronic patient records have been studied at a major hospital in Florida [24]. Of 100 nurses, one-third felt that electronic patient records resulted in a reduced workload and roughly the same proportion preferred being able to provide documentation at the patient's bedside, but said that this was often made difficult. A quarter thought that documentation was improved and the use of electronic patient records would improve patient safety. Nurses with experience of information technology consistently had more positive views than those without such experience.

In this project, we wanted to study whether decision support as part of an electronic, structured patient record system, together with targeted education, could improve the quality of medical treatment while avoiding negative impact on job satisfaction among users.

## 2. Material and Methods

We performed a prevalence study to quantify the proportion of long-term patients with atrial fibrillation that were being given warfarin, were taking neuroleptics, and had not been weighed for the last 30 days. Then, a before-after intervention study was done. The before-after study was conducted as a double cross-sectional design. Instead of randomization, we decided to use the proportion of patients on digitalis, thyroxin, and antidiabetics as internal controls (participants exposed to the same study, but these drugs were not part of any concrete intervention and were unlikely to undergo any changes by external factors.) [25]. The proportion of patients on neuroleptics was in addition followed as a time series (a sequence of data points, measured typically at successive times spaced at uniform time intervals) [26–28] in two nursing homes from 23 October 2008 to 15 July 2010.

### 2.1. Before-Data

The prevalence (before) survey was performed among long-term residents in seven nursing homes (*N* = 513). Bergen, Norway has approximately 250,000 inhabitants and 37 nursing homes (2,300 beds). The number of beds ranges from 20 to 189.

Information about the patients was gathered during the period March-April 2008. Medical students conducted the data collection, which consisted of copying medication cards, weighing by means of a local tool, but standardized clothing, and finding previously recorded weight/date. Electrocardiography (ECG) to diagnose atrial fibrillation was performed and a standardized set of blood samples analysed at the laboratory of the Bethany Hospital. The recorded data were keyed into Excel and the drugs coded according to the Anatomic Therapeutic Chemical (ATC) Drug Register.

The nursing homes were selected based on approximately similar size, function, and public ownership. They were comparable in terms of nursing staff and financing, but some differences existed with regard to other variables (Table 1).

### 2.2. Intervention

After quantifying clinical issues through the before-survey, we installed an electronic, structured patient record system which included decision support. The system had been developed and operating at Løvåsen educational nursing home (developing institution) for several years, funded by the Norwegian Medical Association and the Norwegian Research Council. Installation and training were carried out in the last quarter of 2008, and 6 of the 7 nursing homes were running the system on a daily basis from January 2009 for 12 months. After the active intervention period, the number of nursing homes running the system was reduced to three institutions, owing to contracts between Bergen Municipality and another vendor having been signed before the project started. Løvåsen Nursing Home and two of the study institutions continued and were the only institutions available for follow-up data. Data had to be collected directly from the electronic patient record system.

**Figure 1:**
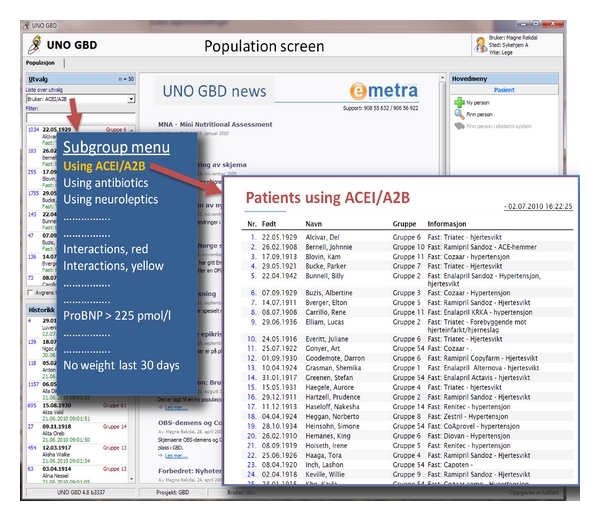
Population filters. How subgroup filters and reports are presented in the UNO GBD patient record system, a structured medical record system with decision-making support, tested in 7 nursing homes in Bergen, Norway 2008–2010. The blue “menu” is for translation purposes.

**Figure 2:**
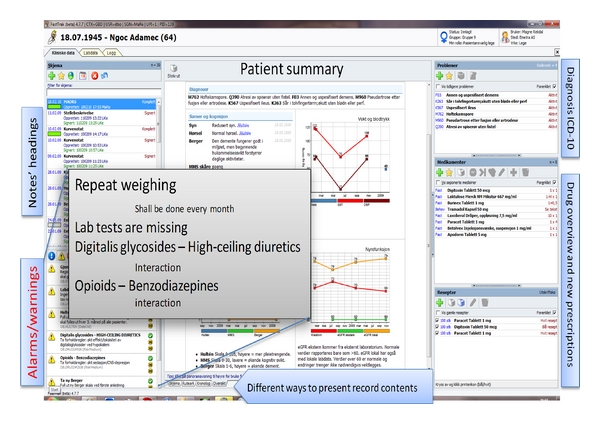
The way “reminders” and interaction warnings are presented in UNO GBD, a structured medical record system with decision-making support, tested in 7 nursing homes in Bergen, Norway 2008–2010. The grey “menu” is for translation purposes.

The technical intervention consisted of population filters and reminders. The system incorporated filters and alarms within several areas, but we decided to test 4 filters and 2 reminders. Population filters were predefined filters that produced lists of patients according to criteria in seconds. The tested population filters were (a) “patients taking neuroleptics” (list and proportion), (b) “patients taking warfarin” (list and proportion), (c) “patients with the diagnosis atrial fibrillation and their treatment status” (list and proportion), and (d) “patients not weighed for last 30 days” (list and proportion). Tested reminders visible in each patient's record, if the criteria were met, were (a) “patient has diagnosis of atrial fibrillation but is not on warfarin” and (b) “patient not weighed for last 30 days.” Figures 1 and 2 demonstrate how this was arranged within the application.

Results from the before-study were presented to the participating doctors during the first 12 months of intervention through three two-hour lectures.

### 2.3. After-Data

Two institutions (E and F in Table 1) participated in the after-study. To collect the data we used the population filters in the medical record system to find the proportions of patients on endpoint drugs, internal controls, and patients not weighed for the last 30 days. Before-data were extracted from the prevalence study for institutions E and F (*N* = 183) for the before-after analysis. Institution E participated with an additional department (22 patients) in the intervention study (*N* = 205, after).

To study user satisfaction, we performed an anonymous survey among the doctors, nurses, assistant nurses, and physiotherapists (*N* = 504). Potential respondents were all users defined in the electronic system. They were invited by e-mail to respond to an electronic questionnaire. 16 questions about position and several aspects of their view on the functionality of the application were presented. Questions were closed, with a score from 1 to 5 (worse–better). The survey was constructed by a group of study nurses from the institutions, with technical organization by the vendor.

### 2.4. Statistics

For the prevalence (before) survey, the recorded data were keyed into an Excel worksheet and the drugs coded according to the Anatomical Therapeutic Chemical (ATC) Drug Register. To compare proportion of populations, Pearson's chi-square test was used. To compare distribution of continuous variables, Kruskal-Wallis' nonparametric one-way analysis of variance was performed. Straight counts were performed in Excel, but JMP 8 was generally used for statistical analyses.

## 3. Results

In the prevalence study, all long-term patients in 7 nursing homes were examined (*N* = 513). There were no dropouts. The before-after study included all long-term patients in two institutions (*N* = 183 before, 205 after), also no drop-outs. 272 (54%) users responded to the questionnaire, Tables 2 and 3.

### 3.1. Before-Survey

In the prevalence study of 484 long-term patients ECG-ed, atrial fibrillation was found in 91 (18.8%). No significant differences existed between the 7 institutions. Of 91 atrial fibrillation patients, 14.2% were anticoagulated with warfarin, that is, 2.5% of all patients. 

72.6% of all patients were not weighed for the last 30 days. There was an average of 121 days between weight measurements. Significant differences were demonstrated between nursing homes. 

Of all patients, 24.4% were taking neuroleptics. 30.0% of the patients taking neuroleptics were on more than one drug.

### 3.2. Before—After

At the two institutions (E and F) participating in the before-after study, the proportion of patients taking neuroleptics was reduced from 33.0% to 21.5% (*N* = 183 before/205 after, chi-square test, *P* = 0.015), that is, a difference of 11.5% (95% CI: 2.3 to 20.6%). Warfarin increased from 3.0% to 9.8% (*P* = 0.013), that is, a difference of 6.8% (95% CI: 1.6 to 12.1%). Use of digitoxin did not increase significantly (8.0% versus 8.5%; *P* = 0.1), thyroxin was not reduced (10.0% versus 8.6%, *P* = 0.765), and antidiabetics did not increase (10.0% versus 10.5%; *P* = 0.996), Figure 4. The proportion of patients not weighed for the last 30 days was reduced from 72.6% to 16.0% (*P* < 0.001), that is, a difference of 56.6% (95% (CI: 47.5 to 64.5%).

**Figure 3:**
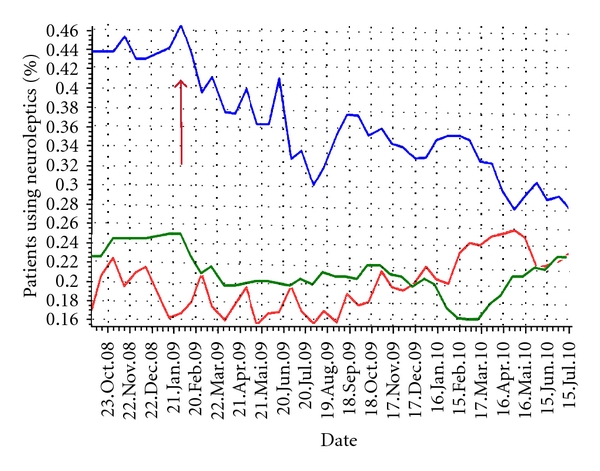
Time-series on the proportion of patients using neuroleptics, in 3 nursing homes in Bergen, Norway 2008–2010, before and during implementation with a structured medical record system with decision support. Blue line represents institution E (see Figure 1), green line institution F, and red line the developing institution. Institution E and F were included in the before-after study. Red arrow indicates implementation startup.

**Figure 4:**
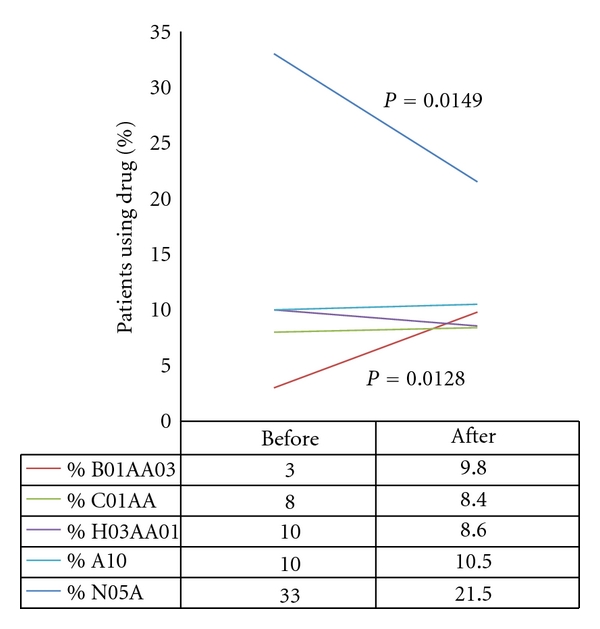
Changes in endpoints and internal controls in the before-after intervention study, among long-term patients in 2 nursing homes in Bergen, 2008–2010. Before and after intervention with a structured medical record with decision-making support, the proportion of patients on the drugs was measured (*N* = 182 before/205 after, chi-square test) Dark blue = Neuroleptics, red = warfarin, others = internal controls.

The time-series for proportions of patients using neuroleptics is presented in Figure 3. 

In the user survey (*n* = 272, 54%), 43% reported great or slightly better job satisfaction. Further results from the user survey are presented in Table 3.

## 4. Discussion

Endpoints changed significantly during intervention by increased use of warfarin, decreased use of neuroleptics, and a higher weighing rate. Job satisfaction was not adversely affected.

### 4.1. Limitations of the Study

The role of nursing homes in the delivery of social and health care services differs between, as well as within, countries. Nursing homes in many countries are managed as part of social care. In Norway, nursing homes are regulated as a health care service. These differences may influence the health issue landscape and composition of staff. Thus, comparisons and generalizations based on our findings should be made with care.

The methods for evaluating change and improvement strategies are not well described. Such study designs should generally be used in a context where they build on appropriate theoretical, qualitative, and modelling work, particularly in the development of appropriate interventions [29]. We feel that we have properly documented the fields in need of improvement and thus study objectives.

Baseline and follow-up data for the first cross-sectional sample were collected, by the prevalence study (before) and (in the case of the second sample) after the intervention, respectively. As it was not possible to collect follow-up data for everyone included in the first study (for political reasons) and the patients included in the second sample were not identical to those in the first due to deaths, the two samples were compared using methods for comparing unpaired data. This is a weakness, but to compensate for the 40% or so annual death rate in nursing homes, the sample size would have had to be much higher to be able to use patients as their own controls, and this may not have been advisable when testing front-end technology in the first study.

Our design must be seen in light of the immaturity of research in nursing homes in general. As far as we know, neither recent data exist on the prevalence of atrial fibrillation in a nursing home population and the use of warfarin, nor is information available on how well basic procedures like weighing are conducted. More information has been available on the use of neuroleptics. There was therefore a need to do a prevalence study, to define valid endpoints, and to do an intervention study. It may be claimed that we should have separated the tasks, but nursing homes are in great need of improvement strategies and time is short before the elderly wave is set to impact heavily on nursing home demands. Financial and political limitations played a role too, obstructing any possibility of performing a full-scale follow-up study. But conversely, it would have been a challenge, in a randomized trial within one municipality, albeit with 37 institutions to choose from, to avoid study group pollution and the “Hawthorne effect” [29, 30]. Starting out with a comprehensive and expensive randomized study would probably not have been ethical due to all the uncertainty and possible threats from new information technology systems, and hence probably difficult to fund. When we started, we did not know if it was possible to install the application, educate, and prepare for critical daily use in 7 institutions with 500 to 800 users within a timeframe of 4 months. Looking back, this may be regarded as the most convincing result of the project.

### 4.2. Effects of Integrated Electronic Decision Support

We used a design where we evaluated endpoints in a before-after study with internal controls [26–28]. The stability of internal controls throughout the intervention reinforces the internal validity of the study. 

With the technology presented, effects can be measured continuously, Figure 3. However, we cannot conclude the extent to which the technical aspect or “awareness of the performance process” is responsible, through education, for the changes measured. Yet without any performance figures, it is not possible to focus on changing processes, so the two ways of influencing results are not independent factors. 

The technology made it possible to monitor performance without time-consuming traditional studies and made performance feedback possible. This is a relatively new concept in medical research, made possible by structured medical input applicable to automatic and continuous analysis. Consequently, the proportion of patients taking neuroleptics could also be followed using time-series, giving more detailed information about the changing process and strengthening the validity of the effect results. As a critical information element, drugs were punched into each patient record at an early stage of the implementation process and this gave us a sufficient “before-point” on the time-series. We discovered that the significant change in the before-after study was due to only one of the participating institutions and that this institution showed up a clear brake in the curve at the point of intervention startup. 

### 4.3. New Possibilities

We see from the time-series on neuroleptics (time-series are part of the patient record system) that the tool also provides a deeper understanding of the change process. By observing the curves, Figure 3, we learn something about possible variation in the use of neuroleptics during the year. This makes one think that performance feedback, over time, may equal performance around a “total average” [31]. Other authors have concluded that evaluative studies should report on usage patterns and progression of outcomes over time [32]. Very few authors have reported on feedback at institutional level with performance comparisons. At vaccination clinics, however, it has been shown that performance feedback at institutional level can improve quality [33]. Logically, this may be one way of influencing both generalized over- and undertreatment in institutions. Our own research (same material, and publications to come) has shown that differences between comparable nursing homes do exist, significantly. With regard to both the proportion of patients using cardiovascular and psychoactive medications, and weight loss and weighing routines, we found significant differences between the 7 nursing homes participating in the prevalence survey (same study, different publications). We hope to continue our research based on this feedback method to test if this is a way to increase equality and quality among a bigger number of institutions.

We are however convinced of the importance of human attention. Technology is a tool only, without any impact, if managers and practitioners are not reacting to warnings and are not exploiting the population filter function!

## 5. Conclusions

Statistics and management data can be continuously produced through daily bedside work. The structured electronic patient record system with decision support we tested can improve drug treatment and monitoring and better implementation of procedures. It can easily be installed for use in nursing homes. Controlled studies on a broader spectrum of clinical and administrative parameters should be performed.

## Figures and Tables

**Table 1:** Characteristics of the 7 nursing homes in the city of Bergen, Norway, participating (*N* = 513 long-term patients).

Nursing home	A	B	C	D	E	F	G	Total	*P*
N:	83	77	39	85	82	101	46	**513**	
No. of places, total/long/short	108/88/20	84/63/21	66/31/35	90/90/0	107/81/26	131/99/32	64/48/16	**650/500/150**	
Location in city	Suburb	Inner city	Suburb	Suburb	Inner city	Suburb	Suburb		
Doctors' hours per place per week	0.4	0.3	0.5	0.3	0.4	0.4	0.5	**0.4**	
Men, %	33.7	33.8	28.2	26.7	21.0	37.6	30.4	**30.2**	**0.273^1^**

Age, years									
Mean	86.7	85.2	84.7	84.9	88.2	79.6	82.2	**84.4**	**<0.001^2^**
Standard deviation	7.6	10.7	7.5	9.1	6.8	15.7	10.0		

Length of stay, days									
Mean	990.0	901.5	973.1	946.1	1,728.9	1,383.1	1,055.2	**1,171.5**	**<0.001^2^**
Standard deviation	1102.9	1038.9	747.8	849.6	1075.2	1299.9	873.43		

Nondemented *n* = 511, %	20.7	31.6	0.0	29.4	41.5	20.8	19.6	**23.4**	**<0.001^1^**
Deviation %	−2.7	8.2	−23.4	6.0	18.1	−2.6	−3.8		

Suffered stroke *n* = 462, %	32.4	10.2	28.2	35.4	21.5	30.0	15.4	**24.7**	**0.009^1^**
Deviation %	7.7	−14.5	3.5	10.7	−3.2	5.3	−9.3		

^1^Pearson' chi-square test; ^2^Kruskal-Wallis test.

**Table 2:** User survey among employees in 7 nursing homes in Bergen (*N* = 272, 54%).

Sex	%	Age	%	Position	*N*	%
Male	12	<40	34	Nurse	97	37
Female	88	40–54	46	Assistant nurse	91	35
		>54	20	Physician	16	6
				Other	58	22

**Table 3:** Results from the user survey among employees in 8 nursing homes in Bergen, Norway, participating testing the “UNO GBD” electronic patient record system (2008–2010), a structured medical record system with decision-making support (*N* = 272, 54%).

(i) 65%—used application on a daily basis
(ii) 81%—exploited reminders when planned the work
(iii) 90%—documentation requirements were met
(iv) 67%—less time consuming
(v) 43%—increased job satisfaction
(vi) 72%—reminders supported them in doing the job
(vii) 83%—application contributed to safer medication
